# P-482. Evaluating Vitamin B12 Deficiency in People Living with HIV Compared to Metformin Users

**DOI:** 10.1093/ofid/ofae631.681

**Published:** 2025-01-29

**Authors:** Lee Nguyen, Bahman Farihi, Catherine Diamond

**Affiliations:** University of California Irvine, Irvine, California; University of California, Irvine, Irvine, California; University of California, Irvine, Irvine, California

## Abstract

**Background:**

Vitamin B12 is a water-soluble vitamin critical for the development and function of the central nervous system, as well as the formation of red blood cells and DNA synthesis. Both HIV and metformin use have been associated with vitamin B12 deficiency. The degree of vitamin B12 deficiency in HV has not been quantified compared to other known causative conditions. Our study aims to describe the incidence of vitamin B12 deficiency in HIV-infected individuals compared to metformin-induced vitamin B12 deficiency. Our secondary goal is to describe the demographics of HIV patients requiring B12 replacement therapy.

**Methods:**

This database study evaluated de-identified medical records from 01/01/2015 to 07/01/2023 at a single large academic institution. We evaluated two study populations: HIV patients and metformin users. For inclusion, both populations required a lab test for vitamin B12 level after starting metformin or the diagnosis of HIV. We defined vitamin B12 deficiency as starting B12 replacement therapy within 4 months of the lab test.

**Results:**

We identified 3,253 HIV and 50,751 metformin-use patients for our study. Among all HIV patients, 717 received a B12 lab test, and 144 (20%, 95% CI 0.173 to 0.232) required replacement therapy. Metformin users had 7773 individuals that received a B12 lab test, and 1187 (15%, 95% CI 0.145 to 0.161) required replacement therapy. The rate of B12 deficiencies was higher among the HIV population (20%) than the metformin (15%), p=0.0007. The relative risk for vitamin B12 deficiency among the HIV population is 1.315 (95% CI 1.126 to 1.536). The number of HIV patients not on metformin with a B12 lab test was 631, and 124 required replacement therapy (19.7%). The HIV population was primarily men (84%), with 50% in the age range of 40-60 years and 63% identified as white.

**Conclusion:**

Vitamin B12 deficiency is a known problem associated with metformin administration and HIV infection. This study identified that our HIV population has higher rates of vitamin B12 deficiency than the metformin group. The cause of this deficiency is unknown. Improved awareness of vitamin B12 deficiency in HIV patients may facilitate improved monitoring and supplementation, which in turn may decrease HIV-associated comorbidities such as dementia and neuropathy.

**Disclosures:**

**Lee Nguyen, PharmD, APh, BCPS-AQ ID, BCIDP**, Merck & Co: Grant/Research Support

